# Cerebellar tDCS does not improve performance in probabilistic classification learning

**DOI:** 10.1007/s00221-016-4800-8

**Published:** 2016-10-20

**Authors:** N. Seyed Majidi, M. C. Verhage, O. Donchin, P. Holland, M. A. Frens, J. N. van der Geest

**Affiliations:** 1 Department of Neuroscience (Ee1202), Erasmus MC, P.O. Box 2040, 3000 CA Rotterdam, The Netherlands; 2 Department of Biomedical Engineering, Zlotowski Centre for Neuroscience, Ben-Gurion University, Beer-Sheva, Israel; 3 Erasmus University College, Rotterdam, The Netherlands

**Keywords:** Learning, Cerebellum, Frontal cortex, tDCS, Cognition

## Abstract

In this study, the role of the cerebellum in a cognitive learning task using transcranial direct current stimulation (tDCS) was investigated. Using a weather prediction task, subjects had to learn the probabilistic associations between a stimulus (a combination of cards) and an outcome (sun or rain). This task is a variant of a probabilistic classification learning task, for which it has been reported that prefrontal tDCS enhances performance. Using a between-subject design, all 30 subjects learned to improve their performance with increasing accuracies and shortened response times over a series of 500 trials. Subjects also became more confident in their prediction during the experiment. However, no differences in performance and learning were observed between subjects receiving sham stimulation (*n* = 10) or anodal stimulation (2 mA for 20 min) over either the right cerebellum (*n* = 10) or the left prefrontal cortex (*n* = 10). This suggests that stimulating the brain with cerebellar tDCS does not readily influence probabilistic classification performances, probably due to the rather complex nature of this cognitive task.

## Introduction

Probabilistic classification learning (PCL) tasks make use of cues that are variously predictive of class membership. For example, people can learn that certain cues are more often associated with category A than category B, despite the fact that no exclusive relationship exists between cue and category. Several neuroimaging studies have suggested involvement of the left prefrontal cortex in PCL tasks (Aron et al. 2004; Flanery 2005). Recently, an fMRI study showed that PCL tasks also induce activation in the right cerebellum as well as in the left orbitofrontal cortex, which increased as a function of the predictive value of stimuli (Lam et al. 2013).

The cerebellum is increasingly thought to be involved in both motor and non-motor functions (Stoodley and Schmahmann 2009; Timmann et al. 2010). Reciprocal cerebro-cerebellar connections connect the cerebellar hemispheres to various parts in the contralateral hemispheres of the cortex (Ramnani 2006; Strick et al. 2009). The general idea is that the anterior cerebellum, via the connections to the motor cortex, supports the cortex in learning new and modifying existing motor behavior (Ito 2000). The connections between the posterior cerebellum and prefrontal cortex suggest that the cerebellum also plays a supportive role in learning cognitive behavior (Balsters and Ramnani 2011; Hayter et al. 2007). For both motor and cognitive learning tasks, it has been observed that stimulating the cerebellum noninvasively using transcranial direct current stimulation (tDCS) affects task performance and task learning (Ferrucci and Priori 2014; Jacobson et al. 2012).

Here we studied the role of the cerebellum in cognitive learning, by assessing the effects of tDCS on performance in the weather prediction task. In this task, which is about learning probabilistic associations between cues and two weather categories, tDCS stimulation of the left prefrontal cortex (Fp3) allegedly improved task performance (Kincses et al. 2004; Nitsche et al. 2007). We hypothesized that tDCS over the cerebellum also induces changes in performance in this PCL task. We predicted that stimulation of either the left prefrontal cortex or the right cerebellum would both result in improved learning in the weather prediction task, as indicated by a reduction in errors and a decrease in response times over the course of the experiment.

## Materials and methods

### Participants

Thirty participants gave their informed consent to participate in this study, which is approved by the local ethical board. Thirteen of the participants were females, and 17 were males. Ages ranged between 19 and 32 years [mean (*M*) = 26.5 years, standard deviation (SD) = 3.4 years]. All participants fulfilled the following criteria: right handedness, normal or corrected-to-normal vision, no metallic implants in or near the head, no electronic implants, no history of neurological deficits and no history of chronic drug abuse. They were recruited through internet advertising; as a motivation, the best performing participant received 30 euros.

Subjects were randomly assigned to one of the three groups of 10 participants each: the anodal cerebellar group (5 women, 5 men), the anodal prefrontal group (3 women, 7 men) and the sham group (5 women, 5 men). All procedures performed were in accordance with the ethical standards of the institutional research committee and with the 1964 Helsinki Declaration and its later amendments.

### Task and stimuli

Subjects performed a variation of the weather prediction task (Gluck et al. 2002) developed in Processing (version 2.0, available at http://www.processing.org). The experiment was performed on a laptop with a 15-in screen with full HD resolution (1920 × 1080 pixels) with cabled computer mouse.

In the experiment, participants were presented with 500 trials. In each trial, a visual stimulus was shown. For each stimulus, the participant had to indicate whether they thought the stimulus predicted sun or rain, based on their experiences in previous trials. They gave their response by clicking with the mouse on the corresponding symbol presented beneath the stimulus. After the response, the correct answer was presented.

The stimulus consisted of the combination of one, two or three distinct cards. In total, there were four distinct cards containing four distinct geometric forms: a circle, a triangle, a square and a diamond. Each card had a height of 200 pixels and a width of 200 pixels. The presence of each individual card was associated with one of both outcomes (sun or rain) with a fixed probability (Gluck et al. 2002). The circle card was associated with rain in 75.6 % of the trials in which a circle was part of the stimulus, for the diamond card this was 57.5 %, for square this was 42.5 %, and for triangle this was 24.4 %. Over all trials, each of the four individual cards was used roughly the same number of times. This implied a specific probability for a stimulus, i.e., a combination of cards, to be associated with an outcome. The frequency of the stimuli and the probabilities of association with rain are shown in Table 1.

**Table 1:** Probability structure of the weather predication task

Type	Stimulus	*n*/100	Rain	Sun	*P* (rain)	Optimal response
A		14	2	12	.143	Sun
B		8	3	5	.375	Sun
C		9	1	8	.111	Sun
D		8	5	3	.625	Rain
E		6	1	5	.167	Sun
F		6	3	3	.500	None
G		4	1	3	.250	Sun
H		14	12	2	.857	Rain
I		6	3	3	.500	None
J		6	5	1	.833	Rain
K		3	1	2	.333	Sun
L		9	8	1	.889	Rain
M		3	2	1	.667	Rain
N		4	3	1	.750	Rain
Total		100	50	50		

Each of the 14 stimulus types (A–N) is a unique combination of 1 to 3 distinct cards from a set of four cards (circle, diamond, square or triangle). For each trial, the order of the cards within the combination was randomized. The column “*n*/100” denotes how many times a stimulus type occurred during a block of 100 trials. The columns labeled “rain” and “sun” denote how often the stimulus is combined with the outcome rain or sun, respectively. The column labeled “*P* (rain)” is the probability that the weather outcome was “rain” for a given stimulus type. The probability that the weather outcome was “sun” is 1 minus *P* (rain). For example, stimulus type G consisted of a diamond, a square and a triangle card and was shown 4 times in a block of 100 trials; it was associated with an outcome “rain” only once, so the optimal response for the participant would be to say that this stimulus predicted “sun”

### Transcranial direct current stimulation (tDCS)

tDCS was delivered by a DC stimulator (NeuroConn GmbH, Ilmenau, Germany). The two 5 × 7 cm^2^ electrodes were placed in synthetic sponges, which were soaked in a saline solution. Two electrode montages were used. In all participants of the prefrontal group, the anodal electrode was placed over the left prefrontal cortex (Fp3) and the reference (cathodal) electrode was placed over the right supraorbital region (on the forehead). In all participants of the cerebellar group, the anodal electrode was placed on the right cerebellar hemisphere (3 cm lateral to the inion) and the reference electrode was placed on the right buccinator muscle. In the sham group, each montage was used in half of the participants. In the prefrontal and cerebellar groups, current was applied for 20 min with an intensity of 2.0 mA (Ferrucci et al. 2008). In the sham group, current was only applied for 30 s to give participants the same tingling sensation as in the other groups. None of the subjects could distinguish the stimulation conditions. In all three groups, a gradual ramp-up (fade in) and ramp-down (fade out) of the current in 30 s reduced unpleasant side effects. Stimulation was started at the beginning of the experiment.

### Questionnaire

After the test, participants had to rate the contribution of each of the four cards to the prediction of sun and rain on a scale from 1 (indicating a high contribution to sun) to 10 (indicating a high contribution to rain). They also had to rate their confidence in this rating on a scale from 1 (not sure at all) to 10 (very sure).

### Design and procedure

Participants were first informed on the safety of the tDCS procedure and the general procedure of the session. After giving consent, the tDCS montage was created and stimulation was applied. Five minutes after the beginning of tDCS stimulation, the experiment started. First, participants received the following literal on-screen instructions:

Predict the weather: Rain or Sun? In this game you will learn to predict the weather by using the cards below: [pictures of the four cards were shown here]. In each round you will see a combination of these cards. You then decide if this combination predicts ‘rain’ or ‘sun’. First, your score is shown on the screen (maximum: 100, minimum: 0). Then you will see the cards and you will give your prediction by clicking on the ‘rain’ or ‘sun’ button. After clicking, the correct answer will turn green. Occasionally a pause screen will be shown so you can take a short break. Two more things: 1. The order of the cards does not matter, it’s only the presence of a card that matters. For example: ‘Circle Square Diamond’ is the same as ‘Square Circle Diamond’. 2. The best participant will receive 30 euro! [sic].

After the experimenter (NSM) made sure the instructions were understood, he left the room and the participant performed the task.

**Fig. 1:**
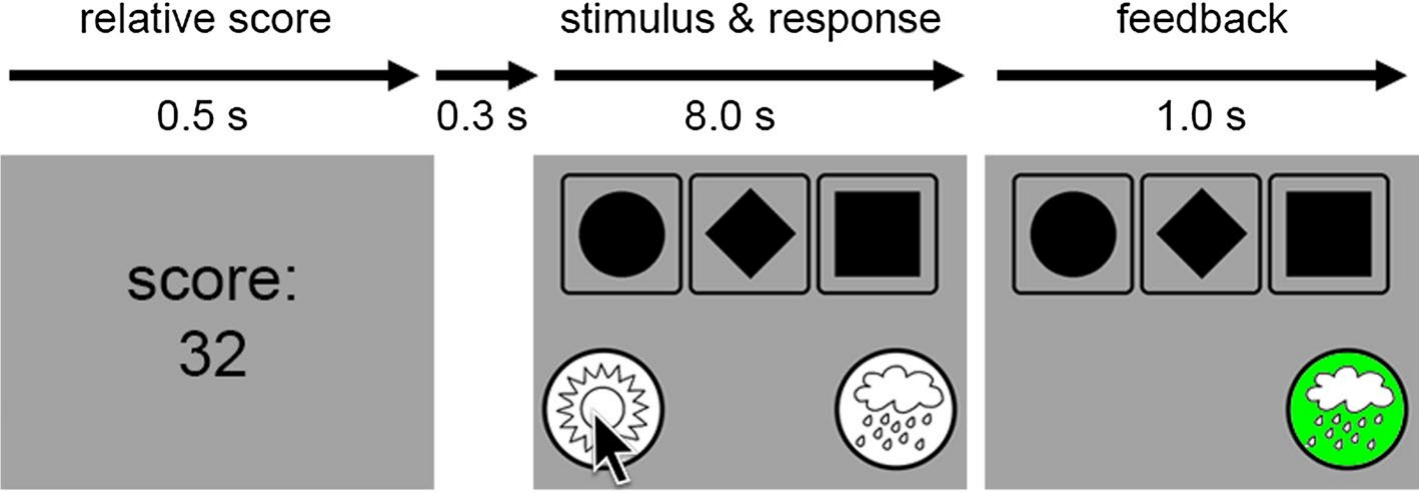
Trial example. After the presentation of the weighted score (weighted average over the last ten trials) followed by a blank screen, a stimulus was presented (a given combination of the four cards, in this example, type N). After clicking with the mouse on one of the weather symbols, the correct answer was presented. In this example, the participant indicated that he believed that this stimulus corresponds to “sun.” In this particular trial, this was not the case, although “sun” was indeed the optimal response for this stimulus, being the correct response in 3 out of 4 presentations (see Table 1)

Before each trial, instead of a fixation cross a weighted score was presented for .5 s. This score was a weighted average of the participant’s performance on the last 10 trials and had a minimum value of 0 and a maximum value of 100. For each trial, this score was calculated with the following formula: Score = 100·∑ (*k* = 1:10) [(11 − *k*)·*C*_*k*_]/55, where *k* runs from 1 to 10, indicating the 10 previous trials. If a correct answer was given *k* trials before the current trial, *C*_*k*_ had a value of 1 (and a value of 0 if it was answered incorrectly). This score was used to keep the participant motivated. After an additional blank screen (presented for .3 s), a stimulus was presented together with a “sun” and “rain” symbol. The participant had a maximum time of 8 s to click on one of the symbols. Feedback was given for 1 s by removing the incorrect symbol and turning the correct symbol green. Finally, the new weighted score was presented, indicating the beginning of the next trial (Fig. 1).

Subjects were presented with 10 blocks of 50 trials each. Pairs of two blocks contained all 100 trials as depicted in Table 1. Stimuli were divided as equally as possible over these two blocks, and we ensured that each block of 50 trials contained 25 stimuli that predicted rain. The order of stimuli was pseudo-randomized. All participants were presented the exact same order of stimuli.

### Data analysis

Performance accuracy was based on the number of optimal responses given by a participant (Gluck et al. 2002). The optimal response for a particular trial is that response that corresponds to the outcome (sun or rain) that is most probable for that stimulus (Table 1). Response times were measured by determining the time between the appearance of the stimulus and the mouse click on the symbol of “sun” or “rain.” Trials with stimulus types F or I were discarded, because they do not have an optimal outcome as the corresponded to rain or sun equally.

For each session, we calculated the percentage of the given responses that were optimal for each block of 50 trials. We also calculated mean response time for each block. In addition, we calculated the percentage of optimal responses and mean response times for the session as a whole.

To examine how participants performed on trials with different predictive values, separate accuracy scores were calculated for high-informative and low-informative trials. High-informative trials consisted of stimulus types A, C, E, H, J and L, because they had very high predictive values for either sun or rain (i.e., *P* (rain) is close to either 0 or 1, see Table 1). Low-informative trials consisted of stimulus types B, D, G, K, M and N, because they had predictive values close to chance (.5).

### Statistical analysis

The effect of tDCS on learning was assessed by a mixed-design ANOVA with one between-participant factor tDCS condition (three levels: prefrontal, cerebellar and sham) and one within-participant factor Block (10 levels: 10 blocks of 50 trials each). In case of sphericity violations, we report corrected estimations of the degrees of freedom. The overall effect of tDCS was assessed by a post hoc one-way ANOVA with tDCS condition as a between-subject factor (three levels: prefrontal, cerebellar and sham stimulation). T-tests were used to compare the performance in high-informative and low-informative trials. Analyses were performed for accuracy scores and for response times separately. Additional analyses were performed comparing the first and last block.

To examine a relationship between speed and accuracy of responses, Pearson correlations between overall accuracy scores and overall mean response times were calculated for each tDCS stimulation condition separately, and for all participants combined.

Interaction effects of tDCS and questionnaire ratings were also assessed by a mixed-design ANOVA with one between-participant factor tDCS condition (three levels) and one within-participant factor cards with four levels (one level per card). All reported values are means ± standard deviations. The threshold of significance was set at 5 percent (*α* = .05).

## Results

### Overall accuracy

On average, participants gave more optimal response across all 500 trials than would be expected by chance (72 ± 9 %, range 55–89 %, *t* (29) = 13.0, *p* < .001, *d* = 4.82). The main effect of tDCS condition on accuracy was not significant (*F* (2, 27) = 2.30, *p* = .12). The post hoc one-way ANOVA showed no significant differences in overall accuracy scores between right cerebellar (67 ± 7 %), left prefrontal (75 ± 11 %) and sham stimulation (73 ± 8 %). Subjects preferred to report “sun” slightly more often than “rain” (52 vs 48 %, respectively; sign test *Z* = 2.23, *p* = .03). The overall accuracy was higher in high-informative trials (79 ± 11 %) than in low-informative trials (57 ± 9 %, *t* (29) = 12.68, *p* < .001, *d* = 2.34).

Subjects performed better over time. Analysis showed a significant effect of blocks (*F* (4.55, 122.80) = 5.95, *p* < .001, *η*_*p*_^2^ = .18, Greenhouse–Geisser corrected, *ε* = .51). This was supported by the fact that, taken together, participants had higher scores in the last block (75 ± 15 %) than in the first block (64 ± 14 %, *t* (29) = 3.59, *p* < .005, *d* = .66).

**Fig. 2:**
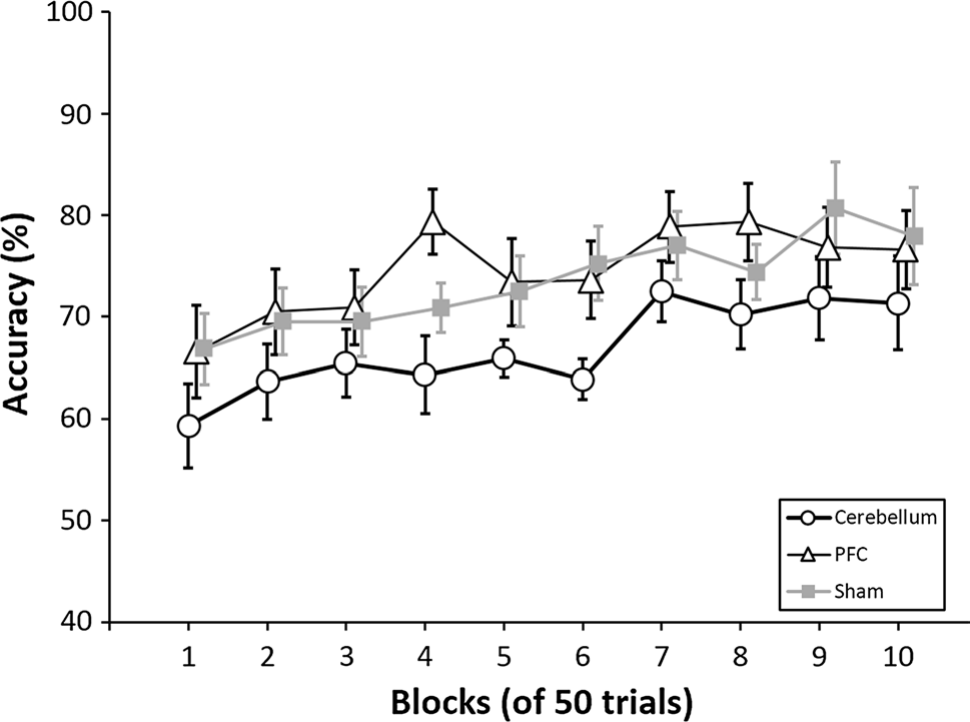
Accuracy. The plot shows the average accuracy scores per block of 50 trials for each of the three stimulation conditions (right cerebellar, left prefrontal or sham). The positive slope indicates that participants performed better over time. No interaction was found between stimulation condition and blocks. *Error bars* represent the standard error of the means

The main effect of tDCS condition was not significant (*F* (2, 27) = 2.30, *p* = .12). Moreover, the interaction between blocks and tDCS condition was also not significant (*F* (9.10, 122.80) = 5.95, *p* = .85), indicating that learning was not influenced by tDCS stimulation (Fig. 2).

### Response times

Mean response times were obtained for each participant over the entire experiment (500 trials). Individual scores varied between .84 and 3.23 s (1.78 ± .50 s). Therefore, the total experiment time varied between 22 min and 42 min which is well within the time limits of 60 min for tDCS stimulation is thought to show an effect (Nitsche and Paulus 2001; Monte-Silva et al. 2013). The main effect of tDCS condition on response time was not significant (F (2, 27) = .78, *p* = .47). The post hoc one-way ANOVA on overall mean response times showed no significant differences between right cerebellar (1.76 ± .54 s), left prefrontal (1.64 ± .58 s) and sham tDCS stimulation (1.93 ± .37 s). Response times for “sun” were the same as for “rain” (1.74 vs 1.79 s, respectively; sign test *Z* = 1.28, *p* = .20). The overall mean response time was lower in high-informative trials (1.67 ± .48 s) than in low-informative trials (1.89 ± .53 s, *t* (29) = 7.13, *p* < .001, *d* = 1.36).

**Fig. 3:**
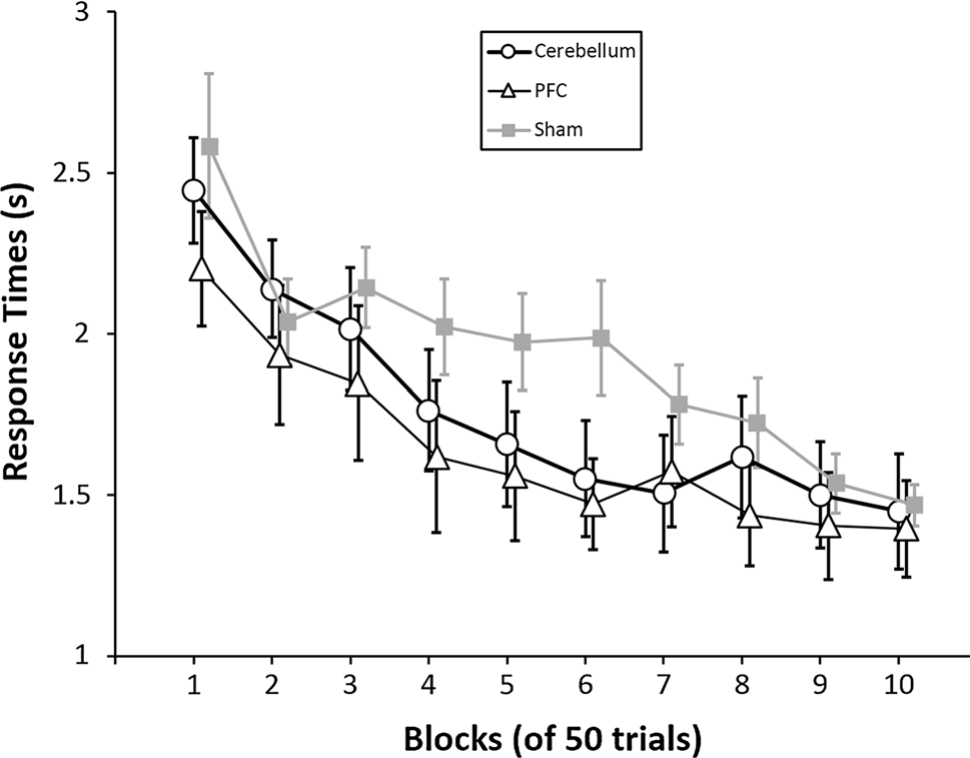
Response times. The plot shows the average response times per block of 50 trials for each of the three stimulation conditions (right cerebellar, left prefrontal or sham). The negative slope indicates that participants performed faster over time. No interaction was found between stimulation condition and blocks. *Error bars* represent the standard error of the means

Subjects responded faster over time. The ANOVA showed a significant effect of blocks (*F* (4.72, 127.40) = 33.30, *p* < .001, *η*_*p*_^2^ = .55, Greenhouse–Geisser corrected, *ε* = .52). This was supported by the fact that participants had lower response times in the last block (1.44 ± .45 s) than in the first block (2.41 ± .63 s, *t* (29) = 9.58, *p* < .001, *d* = 1.83). The main effect of tDCS condition was not significant (*F* (2, 27) = .78, *p* = .47). Moreover, the interaction between blocks and tDCS conditions was not significant (*F* (9.44, 127.40) = 1.78, *p* = .31), indicating that also the decrease in response times across the experiment was not influenced by stimulation (Fig. 3).

We performed an additional analysis including only the first and the last blocks in our ANOVA. This also showed a main effect of block for accuracy *(F*(1, 27) = 12.00, *p* < .01, *η*_*p*_^2^ = .31) and reaction time (*F* (1, 27) = 90.35, *p* < .001, *η*_*p*_^2^ = .77). Main effects of stimulation or interactions between stimulation and block were not significant (all *p* > .32).

No significant correlations between accuracy and response times were found in either of the tDCS conditions (sham: *r* = −.59, *p* = .07; right cerebellar: *r* = .27, *p* = .45; left prefrontal: *r* = −.21, *p* = .56). The correlation was also not seen when all 30 subjects were pooled (*r* = −.14, *p* = .46).

### Questionnaire

**Fig. 4:**
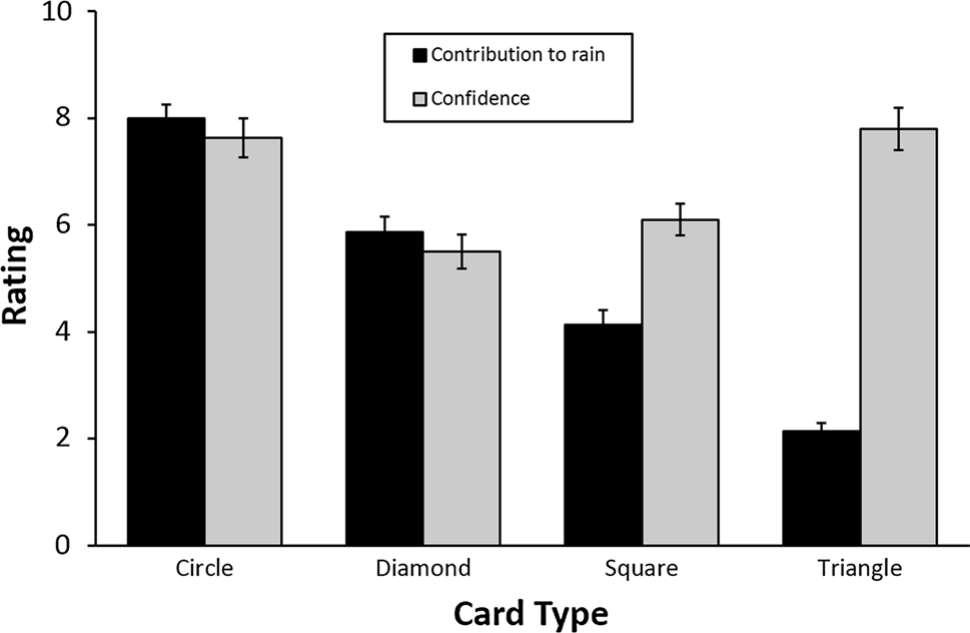
Subjective card contribution and confidence ratings. For each of the cards, participants were asked to rate their subjective contribution to the prediction of rain and the confidence they had in that subjective rating. Contribution ratings follow the same pattern as the actual card probabilities (contributions to rain: *circle* 75.6 %, *diamond* 57.5 %, *square* 42.5 %, *triangle* 24.4 %), indicating that participants had gained knowledge about actual card contributions. Confidence ratings were higher for *circle* and *triangle*, probably due to the high predictive values of these cards. *Error bars* represent the standard error of the means

Ratings of the contribution of each of the four cards to the prediction (Fig. 4) were different for each card (*F* (2.68, 72.48) = 92.07, *p* < .001, *η*_*p*_^2^ = .77, Huyn–Feldt corrected, *ε* = .76). The main effect of stimulation condition and the interaction between card type and stimulation condition were not significant (both *p* > .3). Confidence ratings were also different per card type (*F* (2.93, 79.13) = 16.89, *p* < .001, *η*_*p*_^2^ = .39, Huyn–Feldt corrected, *ε* = .83), but again the main effect of stimulation condition and the interaction were not significant (both *p* > .1).

## Discussion

In this study, we investigated the role of the cerebellum in cognition by assessing the effects of cerebellar direct current stimulation (tDCS) on the weather prediction task, a type of probabilistic categorization learning (PCL). As expected, over the course of the experiment participants learned to improve their performance, showing increased accuracies and reduced response times while the experiment progressed. We only used anodal stimulation over the target brain areas as excitatory effects of anodal stimulation were more profound than the inhibitory effects of cathodal stimulation in a classification task (Jacobson et al. 2012). However, we observed that anodal right cerebellar tDCS stimulation had no effects on either accuracy or response time. Post hoc, we performed additional analyses to investigate the effect of tDCS on the first 50 trials separated in block of 10 trials, similar to previous studies (Kincses et al. 2004; Nitsche et al. 2007). In line with those studies, we did not find a significant interaction between stimulation and blocks. In our view, the lack of such an interaction effect suggests that tDCS had no effect on probabilistic categorization learning. The finding that individual blocks show differences between tDCS conditions in these previous studies does not provide compelling evidence that tDCS affects learning. Moreover, these differences for individual blocks were not replicated here.

Subjects had a preference for the outcome “sun,” which is in line with a previous study (Lam et al. 2013). Contribution ratings followed similar patterns to the card probabilities, indicating participants had gained knowledge of the predictive values of the cards. This is supported by the higher confidence in the high-predictive stimulus types than in the low-predictive stimulus types. These two subjective measures of performance were also not influenced by tDCS stimulation.

The most likely explanation for our results is that the effects of tDCS are too small to have an effect on the performance in the weather prediction task. Moreover, observing such an effect would be hampered by the high variability in performance measures between participants. One way to reduce variability might be accomplished by some kind of normalization, which, on the other hand, also could distort the data. When we normalized our data by subtracting the data of the first block from the subsequent blocks, and performed our analyses again, we observed highly similar outcomes, suggesting again that tDCS is unlikely to have an effect on probabilistic categorization learning.

At first sight, our findings seem to be in contrast with previous studies that did find an effect of anodal prefrontal tDCS (Fp3) using this type of task (Kincses et al. 2004; Nitsche et al. 2007). However, these studies used a total of 50 trials, and effects were only obtained using crude statistical analysis: Both studies did not show, or even test for, interaction effects between blocks and tDCS, but did perform post hoc tests to check for differences per tDCS condition for each block without corrections for multiple testing. Therefore, we think that these previous results do not provide clear evidence for tDCS effects on probabilistic categorization learning yet.

The variability between participants could be due to the fact that the task relies on multiple processes, e.g., working memory, strategy forming and strategy switching, assigning cue combinations to certain outcomes, and visual recognition. A task which relies on less cognitive processes could result in lower variance and would be better for revealing stimulation effects on implicit learning. An example of such a task is the probabilistic guessing task (Hecht et al. 2010). Furthermore, this type of task is more suitable for a within-participant design for stimulation conditions. Here we choose to adopt a between-participant design because we wanted to avoid a test–retest effect due to the possibility that participants would become too familiar dealing with probabilistic rules in our task.

Another explanation for the lack of effect could be that the cerebellum and prefrontal cortex are not critically involved in probabilistic classification. However, the aforementioned neuroimaging studies reported activation of the left prefrontal cortex during probabilistic classification (Kincses et al. 2004; Nitsche et al. 2007). Moreover, it is well established that the left prefrontal cortex is important in many cognitive learning processes. There is therefore a likely role of the left prefrontal cortex in probabilistic classification. Yet, the role of the cerebellum can be debated. Imaging studies suggest that the prefrontal cortex and the striatum are primarily involved with (correct) categorization in a weather prediction task (Seger 2008). This is confirmed by a study investigating PCL in patients with Parkinson’s disease and patients with cerebellar deficits. Results showed that Parkinson but not cerebellar patients are impaired on the weather prediction task, suggesting that successful PCL relies on intact basal ganglia but not on intact cerebellar structures (Witt et al. 2002). Cerebellar activation increases in the right hemisphere with increasing predictive values of card combinations (Lam et al. 2013). Based on these observations, we analyzed the learning curves of the card combinations with a high predictive value alone, in order to increase the change in finding an influence of cerebellar tDCS. We, however, did not find such an effect.

Several adjustments to our experimental design can be suggested as various confounding factors like the exact electrode location, and stimulation intensities and stimulus durations could influence the effect of tDCS (Gluck et al. 2002). The tDCS settings used in our study were found to modulate behavior, albeit in different tasks (Nitsche et al. 2007; Tomlinson et al. 2013). Nonetheless, in order to be able to claim that cerebellar tDCS does not influence probabilistic classification performances at all, future studies could look into the effect of these possible confounding factors, ideally adopting a within-subject design. On the other hand, negative findings with tDCS in cognitive tasks are not unexpected. There is increasing discussion about the robustness of tDCS effects, and publication of negative findings is of importance (Horvath et al. 2015; Antal et al. 2015).

## Conclusion

We were not able to demonstrate the role of the cerebellum in probabilistic classification. Although we cannot rule out that such an effect is present, our data suggest that a tDCS effect on this task is likely to be quite small. Studies utilizing a different task or stimulation technique are needed to investigate cerebellar involvement in complex cognitive learning processes.
